# Colonization of *Serendipita indica* promotes resistance against *Spodoptera exigua* in onion (*Allium cepa* L.)

**DOI:** 10.3389/fmicb.2023.1190942

**Published:** 2023-07-26

**Authors:** Praveen Roylawar, Kiran Khandagale, Satyabrata Nanda, Parakkattu Sulochanan Soumia, Sangita Jadhav, Vijay Mahajan, Suresh Gawande

**Affiliations:** ^1^ICAR-Directorate of Onion and Garlic Research, Pune, India; ^2^Department of Botany, S.N. Arts, D.J.M. Commerce and B.N.S. Science College, Sangamner, Maharashtra, India; ^3^Department of Biotechnology, Centurion University of Technology and Management, Paralakhemundi, India

**Keywords:** onion, *Spodoptera exigua*, *Serendipita indica*, insect herbivory, endophyte-mediated resistance

## Abstract

Plant-endophyte symbiosis influences plant defense and growth. *Serendipita indica* is a root endophyte that promotes growth and induces tolerance against biotic and abiotic stress in plants. In this study, we examined the effect of *S. indica* colonization on herbivore (*Spodoptera exigua*) resistance of onion (*Allium cepa* L.). We found that colonization of *S. indica* in the roots of onion significantly reduced the feeding damage of leaves by *S. exigua* larvae, and also resulted in a reduction in weight gain of the larvae when fed on *S. indica* plants. This enhanced resistance is a result of modulation of antioxidant and defense enzymes/genes in the host by *S. indica* mutualism. Specifically, the activities of enzymes such as Superoxide dismutase, peroxidase, polyphenol oxidase, phenylalanine ammonia-lyase, and H_2_O_2_ content were significantly higher in the early stages of *S. exigua* feeding in the *S. indica* colonized plants compared to the non-colonized counterparts. Similarly, defense genes also showed modulation in response to this tripartite interaction of onion -*S. indica* mutualism and *S. exigua* herbivory. The hierarchical cluster analysis and principal component analysis indicated a clear difference in the onion biochemical responses, which is due to the *S. indica* symbiosis. Our investigation demonstrates that onion-*S. indica* symbiosis significantly decreases chewing injury by efficiently modulating antioxidant and defense enzyme activities and gene expression in response to *S. exigua* herbivory. Therefore, *S. indica* can be used as a potential biocontrol agent for sustainable management of this important pest of Alliums.

## Introduction

Onion (*Allium cepa*) is an important vegetable from the *Allium* genus in the Amaryllidaceae family. Onions are cultivated and consumed worldwide. Onions hold high economic and medicinal significance for their uses in culinary practices and traditional medicines. Quercetin, the major bioactive compound in onion acts as a lachrymatic agent and along with other secondary metabolites provides anti-bacterial, anti-fungal, anti-cancer, and antioxidant activities (Khandagale and Gawande, 2019). On the other hand, allicin, an organosulphur compound in garlic is used as a stimulant, diuretic, diaphoretic, and expectorant (Nanda et al., 2016). India is the second largest producer of onion in the world (FAOSTAT, 2021). However, India cultivates onions in the largest production area of 1.4 lakh million hectare with productivity of 16.4 t/ha (FAOSTAT, 2021). Thus, it is evident that the productivity of onions in India is low compared to the global productivity rates. Severe crop losses due to different pathogen attacks and insect infestations are the major causes of this lower productivity rate in India. Among the insect pests, the Lepidopteran insect *Spodoptera* spp. has been reported to be a notorious pest in onions causing huge crop losses (Fernandes et al., 2012; Soumia et al., 2020; Cokola et al., 2021).

*Spodoptera* is a genus of moths having mostly polyphagous feeding habits. In onions, the infestation of *S. frugiperda* (fall armyworms) and *S. exigua* (beet armyworm) have been reported. *S. frugiperda* is a major pest of some cereals and field crops, including maize, sugarcane, millets, sorghum, and cotton, and causes huge crop damage. However, both recent and earlier reports have confirmed the infestation of *S. frugiperda* on onions in different parts of the world (Fernandes et al., 2012; Cokola et al., 2021). Similarly, *S. exigua* infests vegetable crops and cereals, including cruciferous crops, okra, and wheat. The first report of *S. exigua* infestation in *Allium* spp. (*A. fistulosum*, welsh onion) came in 1993. Kawai et al. (1993) reported that *S. exigua* could invade the leaf blades and the infestation was contagious among the onion hills. Similarly, several subsequent studies reporting the infestation incidents of *S. exigua* on onions confirmed that onion is a host plant for this notorious pest (Zheng et al., 2000; Ueno, 2015; Ilmi et al., 2022). These reports of the host shifting suggest the strong polyphagous and well-diverse host adaptability of the *Spodoptera* spp. Moreover, in India it is emerging pest of onion, recorded a significant foliage damage of 70–80% in *rabi* onions when subjected to infestation by *S. exigua* (Soumia et al., 2020). Therefore, the management and control of *Spodoptera* spp. are crucial in achieving crop security. Currently, the use of synthetic pesticides is the most practiced method of controlling *Spodoptera* infestation. However, the exploitation of these broad-spectrum chemical pesticides has caused the elimination of natural enemies, insect resurgence, and insecticide resistance along with other environmental hazards. Therefore, alternative environment-friendly and sustainable solutions to control pest infestations are required.

*Serendipita indica* (formerly known as *Piriformospora indica*) is a widely-studied plant endosymbiont having phyto-promotional and stress-alleviating roles (Gill et al., 2016). Notably, *S. indica* is a cultivable symbiont with beneficial properties such as growth promotion, induction of stress tolerance etc. The role of *S. indica* in enhancing plant growth, development, and nutrient acquisition has already been reported (Gill et al., 2016). In recent times, the use of *S. indica* as a biocontrol agent in different crops against several biotic stressors is being focused on. Several reports have confirmed the use of *S. indica* as a biocontrol agent in controlling the infestation of both pathogens and insects. For instance, the use of *S. indica* as a biocontrol agent protected Arabidopsis plants from the soil-borne vascular pathogen *Verticillium dahliae*. *S. indica* restricted the growth of *V. dahliae* in Arabidopsis and regulated the Arabidopsis defense responses against the pathogen (Sun et al., 2014). Similarly, Atia et al. (2020) reported that *S. indica* improved tolerance of cucumber plants against the root-knot nematode infection by regulating photosynthesis and innate immune responsive genes. Interestingly, the use of *S. indica* has been explored to control insect infestations, including the *Spodoptera* spp. The colonization of *S. indica* in rice plants improved the resistance against *Cnaphalocrocis medinalis* (rice leaffolder) through the activation of antioxidant- and jasmonic acid (JA)-mediated defense responses (Chen et al., 2022). In sweet potato plants, the colonization of *S. indica* enhanced the JA levels, induced the expression of defense-responsive genes, and increased trypsin inhibitory activity resulting in a significant reduction in the performance of *S. litura* (Li et al., 2021). Therefore, considering these facts, the potential of *S. indica* as a biocontrol agent against *S. exigua* has been investigated in this study. Further, the mechanism of onion defense modulation against the *S. exigua* infestation is explored by analyzing the key defense-related gene expressions, including WRKY transcription factors, and JA and salicylic acid (SA) pathway-related genes. In addition, the antioxidant activities in onions are studied by analyzing the peroxidase (POD), superoxide dismutase (SOD), and hydrogen peroxide (H_2_O_2_) levels. Furthermore, defense enzymes like phenylalanine ammonia-lyase (PAL) and polyphenol oxidase (PPO) were also included in the study. Overall, the findings of this study provide valuable insights into the *S. indica*-mediated control of *S. exigua* infestations in onions.

## Materials and methods

### Plant material and fungal culture

The susceptible onion cultivar “Bhima Super” was used as plant material for this study. The culture of *S. indica* was obtained from Dr. J. Vadassery (National Institute for Plant Genome Research, New Delhi, India) and maintained on potato dextrose agar (PDA) medium.

### *Serendipita indica* colonization of onion plant roots

The cultivation and harvesting of *S. indica* mycelium were done as described in Roylawar et al. (2021). Ten grams of minced mycelium per 100 g of autoclaved soil was mixed in a perforated plastic crate (20′ × 13′ × 11′). Seeds of Bhima Super were sown in these autoclaved soil crates with *S. indica* mixture while, in control, seeds were sown in autoclaved soil only. The colonization of onion roots was examined microscopically after 30 days of sowing in *S. indica*-treated pots. Roots of 10 randomly selected *S. indica*-treated plants were incubated at 90°C in 10% KOH for 5 min. Followed by 1 min. in 1 N HCl and then stained with trypan blue (0.02%) for 60 min. at room temperature. Colonization was observed under a light microscope in 40X magnification (LEICA DM2500).

### Insect material and rearing

The egg masses and larvae of *S. exigua* (Lepidoptera: Noctuidae) collected from the experimental field of ICAR-Directorate of Onion and Garlic Research, Rajgurunagar (Pune, India) (18.8543°N, 73.8876°E) during the *Kharif* season were reared in laboratory under ambient conditions [25 ± 2°C and 65 ± 5% relative humidity (RH); 16:8 h light:dark photoperiod]. Larvae were raised on fresh castor leaves in transparent acrylic jars covered with a piece of muslin cloth as described by Saeed et al. (2017). When the pupae were ready, they were surface sterilized with 0.1% sodium hypochlorite solution and transferred into fresh acrylic jars for adult emergence and mating. Castor leaves were used as the oviposition substrate whereas cotton swabs with 50% honey and water as a diet in adult cages. Insects were sub-cultured consecutively for three generations. Subsequent progenies emerging from the sub-culture were used for the present experiment.

### Onion-*Spodoptera exigua* interaction assays

To study the effect of induced systemic resistance of *S. indica*-treated onion against *S. exigua*, single onion plants potted in individual plastic pots (20 cm diameter) were taken with twelve replications each for *S. indica*-treated and control. For the in-*planta* experiments, artificial infestation with one third instar larvae per plant (eight-week-old *S. indica* treated onion plant) was released under glasshouse conditions. Before the feeding assay, larvae were starved for 6 h. After 24 h of infestation, total leaves and damaged leaves were counted and their overall damage rating is converted to a 0–3 scale (0 = No damage; 1 = up to 1/3 of leaf area scarped; 2 = 1/3 to ½ of leaf area scraped; 3 = more than ½ of leaf area scraped) (Kavitha and Reddy, 2012).


$$\begin{array}{l} \%\mathrm{Rating}\;\left(R\right)=\frac{\begin{array}{l} \left(\mathrm{No}.\mathrm{of leaves with damage grade of}\;1\times 100\right)\\ \;\;\;\;\;\;\;\;\;\;\;\;\;\;\;\;\;\;\;\;\;\;\;\;\;\;\;\;\;\;\;\;\;\;\;\;\;\;\;\;\;\;\;\;\;\times 1 \end{array}}{\mathrm{Total}\;\mathrm{No}.\;\mathrm{of leaves observed}\;}\\ \;\;\;\;\;\;\;\;\;\;\;\;\;\;\;\;\;\;\;\;\;\;\;\;\;\;\;\;\;\;\;\;+\;\frac{\begin{array}{l} \left(\mathrm{No}.\mathrm{of leaves with}\;\mathrm{damage grade of}\;2\times 100\right)\\ \;\;\;\;\;\;\;\;\;\;\;\;\;\;\;\;\;\;\;\;\;\;\;\;\;\;\;\;\;\;\;\;\;\;\;\;\;\;\;\;\;\;\;\;\;\times 2 \end{array}}{\mathrm{Total}\;\mathrm{No}.\;\mathrm{of leaves observed}}\\ \;\;\;\;\;\;\;\;\;\;\;\;\;\;\;\;\;\;\;\;\;\;\;\;\;\;\;\;\;\;\;\;+\frac{\begin{array}{l} \left(\mathrm{No}.\mathrm{of leaves with damage grade of}\;3\times 100\right)\\ \;\;\;\;\;\;\;\;\;\;\;\;\;\;\;\;\;\;\;\;\;\;\;\;\;\;\;\;\;\;\;\;\;\;\;\;\;\;\;\;\;\;\;\;\;\;\times 3 \end{array}}{\mathrm{Total}\;\mathrm{No}.\;\mathrm{of leaves observed}} \end{array}$$

For further biochemical and gene expression analyses, leaf tissue was also collected at 6, 12, and 24 h-post infestations (hpi), powdered in liquid nitrogen, and stored at −80° C.

### Insect feeding assays

The *S. indica*-colonized onion plants were used to conduct the *S. exigua* feeding bioassays (Li et al., 2021). The 3^rd^ instar *S. exigua* larvae were placed on the onion leaves for the feeding evaluations. Both initial and final larval weights (*n* = 15) were determined using an analytical balance after 3 days of feeding and the weight gain was measured.

### Evaluation of H_2_O_2_ content

The level of hydrogen peroxide (H_2_O_2_) was estimated according to the method described by Roylawar et al. (2021). In 4-(2-hydroxyethyl)piperazine-1-ethanesulfonic acid (HEPES) buffer (50 mM; pH 7.5) 50 mg of frozen leaf sample was mixed and centrifuged at 12,000 rpm (4°C) for 5 min. The supernatant (100 μL) was mixed with 1 mL reaction volume containing HEPES buffer (50 mM, pH 7.5) and potassium titanium oxalate (2.5% in 20% H_2_SO_4_). The absorbance was recorded at 410 nm and the H_2_O_2_ content was deduced from the standard calibration curve.

### SOD activity

200 mg leaf tissue from each treatment was homogenized in an ice-cold potassium phosphate buffer (0.1 M, pH 7.2) with ethylenediaminetetraacetic acid disodium salt (Na-EDTA) (0.1 M) and polyvinylpyrrolidone (0.5%). Homogenate was centrifuged for 15 min. (20,000 rpm at 4°C) and the supernatant was used for the estimation of SOD and POD enzymatic activities. Superoxide dismutase activity was measured according to Beauchamp and Fridovich (1971). The assay was performed in a 3 mL reaction volume containing phosphate buffer (50 mM, pH 7.8), riboflavin (50 μM), methionine (20 mM), EDTA (1 mM), nitro blue tetrazolium chloride (NBT) (1 mM), and 70 μL enzyme extract. The reaction mixtures were prepared in two replicate sets for each treatment. One set was kept in the dark and the other was incubated in the light for 20 min. Under 100 μE m^−2^ s^−1^ light intensity at 26 ± 2° C. Absorbance was measured at 560 nm, and the dark-incubated reaction mixtures of each sample were used as a blank.

### POD activity

The POD activity was measured as described by Xu et al. (2011). The assay was performed in a 3 mL reaction mixture containing phosphate buffer (0.1 M, pH 7.2), guaiacol (30 mM), H_2_O_2_ (20 mM), and enzyme extract. The POD activity was determined using the molar extinction coefficient of 26.6 mM^−1^ cm^−1^ and expressed in Ug^−1^ fresh wt.

### PPO activity

For PPO activity measurement, 400 mg frozen leaves were homogenized in ice-cold 1 mL of 50 mM phosphate buffer (pH 7.1) containing 0.1% (w/v) SDS. The homogenate was centrifuged at 10,000 g for 15 min. at 4° C and the supernatant was collected. The protein concentration of the enzyme extract was determined by the Bradford method (Bradford, 1976). The activity of PPO was determined spectrophotometrically at a wavelength of 400 nm in a reaction mixture (1 mL) containing 50 mM phosphate buffer, pH 5.5, 10 mM 3-methyl-catechol, and 150 μL enzyme extract. The assay buffer was aerated for 5 min before use. Catalase (280 Units ml^−1^; Sigma) was added to remove any H_2_O_2_ present and prevent interference by peroxidases (Sherman et al., 1991). In addition, the specificity of the assay was verified by performing parallel assays with 1 mM kojic acid, a specific PPO inhibitor. Enzyme activity was determined according to Cheema and Sommerhalter (2015). One unit of PPO activity was defined as the amount of enzyme producing 1 μmol of quinone per minute at 30° C. Specific activity was expressed in U/mg protein.

### PAL activity

The activity of PAL was measured as reported by Roylawar et al. (2021). For enzyme extraction, 1 g of each leaf sample was ground in liquid nitrogen and homogenized in 3 mL of cold Tris–HCl buffer (50 mM, pH 8.0), containing 2-mercaptoethanol (1.5 mM) and 0.5% polyvinylpyrrolidone (PVP), then centrifuged at 12,000 rpm for 10 min. at 4° C. The resultant supernatant was used as an enzyme extract for the PAL assay. The assay was performed in a 3 mL reaction mixture containing 1.5 mL of Tris–HCl buffer (50 mM, pH 8.0), 200 μL of L-phenylalanine (1.5 mM), 0.9 mL of distilled water, and 0.3 mL of enzyme extract. The reaction mixture was incubated at 30 ± 1° C for 90 min., and then 1 mL of HCl (2 N) was added to stop the reaction. Further, 3 mL of toluene was added to the mixture before centrifugation at 14,000 rpm for 10 min. at 4° C. The upper layer (enzyme-toluene mixture) was removed carefully and read at 290 nm. Toluene (in the absence of the enzyme) was used as a blank. A standard curve for trans-cinnamic acid (0–100 μM) was plotted to deduce the quantity of cinnamic acid formed in the sample.

### Defense gene expression analyses

#### RNA isolation and cDNA synthesis

The leaf samples were collected at 12 and 24 hpi from all treatments. Three plants pooled as one biological replicate and two such biological replicates were incorporated into the study. Total RNA was isolated from the leaves of two biological replicates using the RNeasy Plant Mini Kit (Qiagen, Germany). The potential genomic DNA contamination was eliminated with Dnase I (Fermentas, Lithuania) treatment. RNAs were quantified using NanoDrop (ND1000 Spectrophotometer, ThermoFisher Scientific, United States). First-strand cDNA was synthesized by reverse transcription from 1 μg RNA using a revert aid first-strand cDNA synthesis kit (Fermenats, Lithuania). cDNAs were stored at −80°C until used in further study.

#### RT-qPCR analyses

Gene sequences of onion available at NCBI were used for primer designing by Gene Runner software. Expression analysis of defense genes was performed in LightCylcer 480 II instrument (Roche, Germany). A 10 μL PCR reaction was carried out in triplicate for each treatment with Light cycler 480 SYBR Green l master mix (Roche, Germany). The PCR cycling was programmed as follows: initial denaturation at 95°C for 5 min, 40 cycles of 95°C for 15 s, 55–60°C for 45 s, and 72°C for 30 s. The melting curve analysis was done to confirm PCR specificity. The onion *Actin* gene (*AcActin*) was used as the internal control for the normalization of cycle threshold (CT) value and relative transcript abundance was calculated according to the 2^−∆∆Ct^ method (Schmittgen and Livak, 2008). The details of primers used in the present qPCR analyses are depicted in Table 1.

**Table 1:** Selected genes and respective primer set used for gene expression analysis from onion leaves.

Gene description	NCBI ID	Primer sequence (5′-3′)	Size (bp)
*AcLOX1*	KU363822.1	F-AGGCACGGCAGTGTTAATGA R-CACCAGCCGCTACAGATGAT	204
*AcGST*	AB300334.1	F-TCGTGAGAGTGATTGCGGTT R-CGCAGGTAGGAGGTCTGTTC	210
*AcPAL*	KF421110.1	F-AGGTGGAAGTTGTAAGGGCG R-CCATTGCAAACCGCCTCAAA	187
*AcWRKY11*	JR859298.1	F- TACGCTCAACGATAGCTCCG R- GCCGGAACCATTATCCTCCA	208
*AcWRKY70*		F-TGGAGAGTTCGCTGGTCAAA R-TACAGCCTCTGCGAGAAACG	210
*AcbHLH*	KY273104.1	F-CATCCAAAGCCTGCCCTCTC R-TATCTGCTGGCGTTATGGGTG	226
AcTI	AB083109.1	F-AATGCTTGGTGTCTTGGCTG R-GTGGAGGATTCAGGTCGAGG	212
*AcActin*	GU570135.2	F-GCACCAAGAGCAGTATTC R-CCAAATCTTCTCCATGTCA	183

Sequence for WRKY11 was obtained from the Onion genomic resource database (http://webtom.cabgrid.res.in/ogr). WRKY70 sequence is obtained from Ghodke et al. (2020).

### Statistical analysis

The gene expression data were analyzed by two-way ANOVA at *p* ≤ 0.05 to evaluate the statistical significance. Similarly, the principal component analysis (PCA) and hierarchical clustering analysis (HCA) of H_2_O_2_, SOD, POD, PAL, and PPO activity were performed on the MetaboAnalyst 5.0 platform (Pang et al., 2021). The PCA was carried out to compare onion plant response to *S. exigua* infestation under control conditions, with or without *S. indica* treatments. The HCA was employed to evaluate the similarities and dissimilarities between the response variables. The clustering was done by using the Euclidean distance and Ward clustering algorithm. All data sets were normalized before they were used for PCA and HCA.

## Results

### *Serendipita indica* colonization in the onion root and efficacy against *Spodoptera exigua*

**Figure 1:**
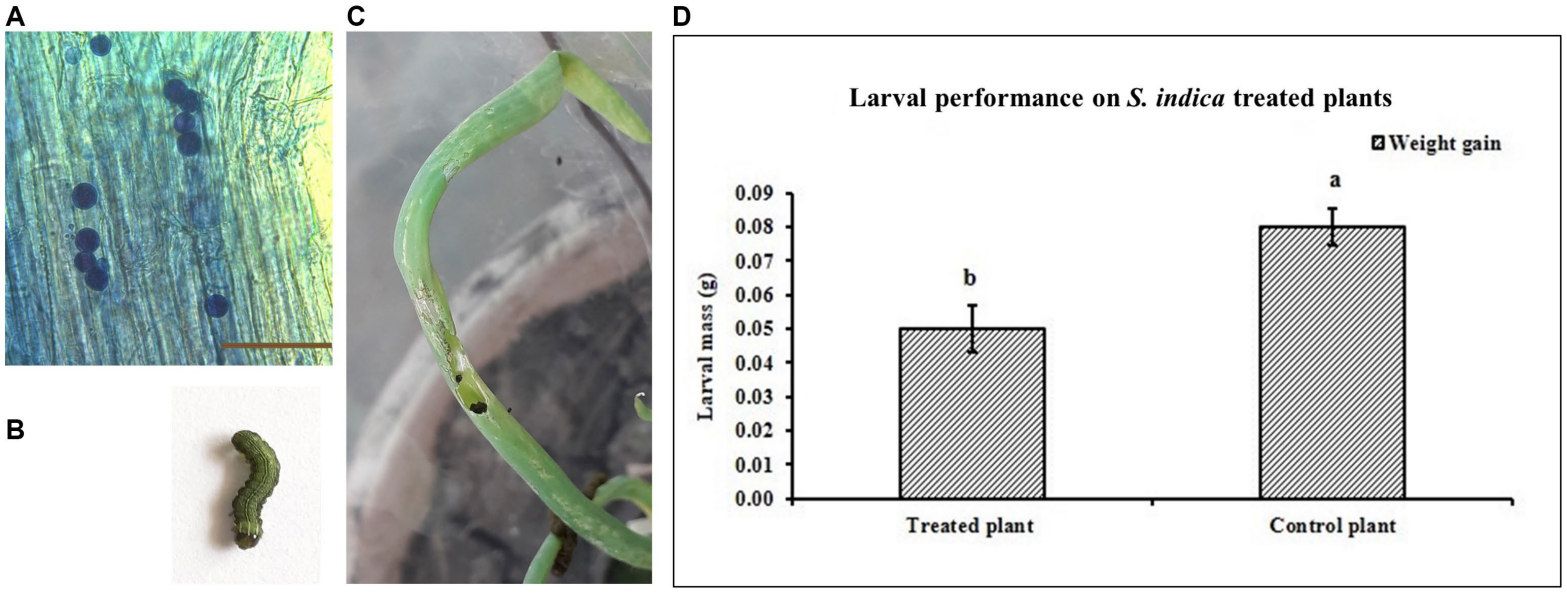
**(A)** Colonization of onion root by *S. indica*. **(B)** 3rd instar larva of *S. exigua*. **(C)** Leaf damaged due to feeding of *S. exigua*. **(D)** Performance of the *S. exigua* larvae on the *S. indica*-treated and untreated (control) plants. Data are represented as mean ± SE and the alphabets indicate the statistical significance computed at *p* < 0.05.

Colonization of onion root by *S. indica* was confirmed after 30 days post inoculation (DPI) by microscopy. Pear-shaped chlamydospores were observed in the onion root cortex (Figure 1A). In control plants there was no colonization observed. The efficacy of the *S. indica* treatment in controlling *S. exigua* was evaluated by checking the performance of *S. exigua* larvae (Figures 1B,C). The results suggested that in the treated plants, the insect larvae performance was reduced as compared to the control plants (non-treated). The larval weights were significantly less on the treated plants as compared to the larvae fed on the non-treated plants (Figure 1D). The chewing injury by *S. exigua* larvae in *S. indica* colonized plants was significantly reduced compared to non-colonized plants (Supplementary Table S1).

### Quantification of H_2_O_2_

**Figure 2:**
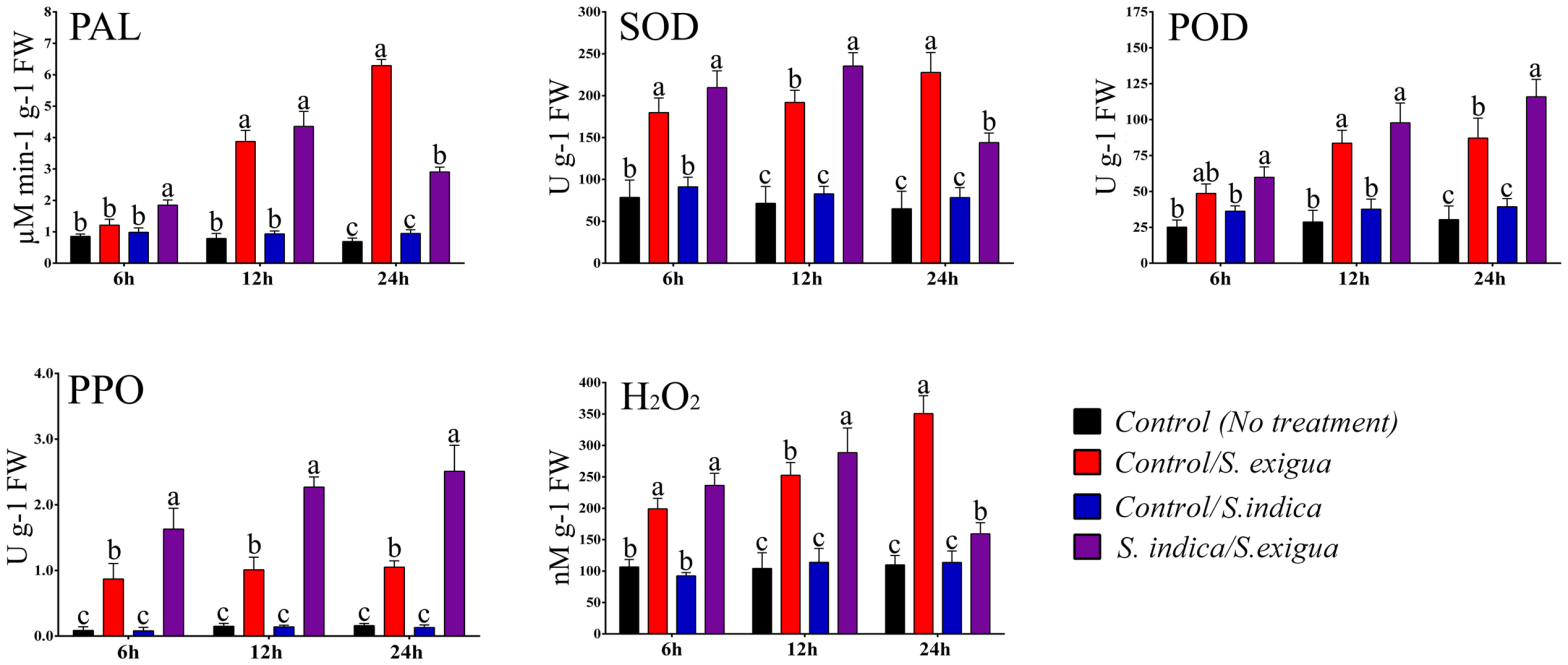
The dynamics of different stress-responsive enzyme activities and H_2_O_2_ levels in different onion plant groups. Data are represented as mean ± SE and the alphabets indicate the statistical significance computed at *p* < 0.05.

The H_2_O_2_ levels were measured at 6, 12, and 24 h post infestation (hpi). No significant increase in the H_2_O_2_ levels was observed at 6 hpi in control plants and *S. indica* colonized plants in response to *S. exigua* infestation. Whereas, at 12 hpi *S. indica*-colonized plants showed an 18.55% increase in the H_2_O_2_ level as compared to non-colonized plants in response to *S. exigua* (Figure 2). Furthermore, in the late stage of infestation, the level of H_2_O_2_ was highly reduced in *S. indica*-colonized plants, while the non-colonized plants showed a 55.73% increase in H_2_O_2_ accumulation. There was no significant difference observed in control and *S. indica*-colonized uninfested plants.

### *Serendipita indica* primed temporal modulation of activities of stress-responsive enzymes

We have assessed the enzymatic modulation of selected stress-responsive enzymes at 6, 12, and 24 hpi in uninfested and infested plants. As observed in the plots (Figure 2) *S. indica-*primed *S. exigua*-infested plants showed an enhanced temporal modulation of the enzymes. In the case of SOD, the enzymatic activity can be very well correlated with the accumulation of H_2_O_2_. In the early stage of *S. exigua* infestation comparatively, there was no significant change in the level of SOD and POD in control plants and *S. indica* colonized plants. Compared to the uninfested plants, *S. exigua* infestation significantly triggered the enzymatic response in both primed and unprimed plants at all-time points. At 12 hpi *S. indica-*primed plants showed 18.41% more expression of SOD upon *S. exigua* infestation compared to control-infested plants. However, the level of SOD was significantly decreased by 17.69% at 24 hpi in *S. indica* primed plants compared to control plants upon *S. exigua* infestation. The enzymatic expression of POD was insignificant at 12 hpi however, the expression significantly enhanced by 24.77% at 24 hpi in *S. indica-*primed plants compared to control plants upon *S. exigua* infestation.

The other stress-responsive enzymes like PAL and PPO showed 31.53 and 46.62% significantly enhanced expression at 6 hpi in *S. indica-*primed plants compared to control plants upon *S. exigua* infestation. Moreover, at 12 hpi PAL did not show any significant difference however, PPO showed 55.50% enhanced expression in *S. indica-*primed plants compared to control plants upon *S. exigua* infestation. Furthermore, the level of PAL has significantly decreased at 24 hpi by 53.8% while, PPO expression was significantly enhanced by 58.16% in *S. indica* primed plants compared to control plants upon *S. exigua* infestation. We have noticed that level of PAL was significant in an early phase of infestation while PPO was significantly higher at all studied time points. In uninfested plants, we did not observe any significant changes throughout the study indicating that together with antioxidants enzymes PAL and PPO are actively triggered in response to *S. exigua* infestation. It is worth mentioning that the major significant changes were observed at 12 and 24 hpi after *S. exigua* infestation.

### PCA and HCA analysis

**Figure 3:**
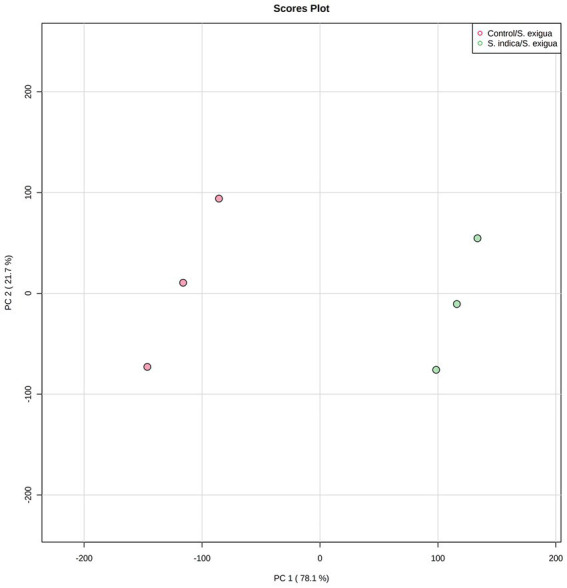
PCA analysis of the onion responses variable to *S. indica* treatment and *S. exigua* infestation.

The H_2_O_2_, SOD, POD, PAL, and PPO activities in the control (without *S. indica* treatment) and test (with *S. indica* treatment) were compared by PCA under *S. exigua* infestation. The data from three time points, including 6, 12, and 24 hpi were collected and used in the PCA. A score plot was plotted between PC1 and PC2, the principal components accounting for 99.8% of the total variance (Figure 3). On the score plot, the *S. indica*-treated samples and the non-treated samples were well-separated.

**Figure 4:**
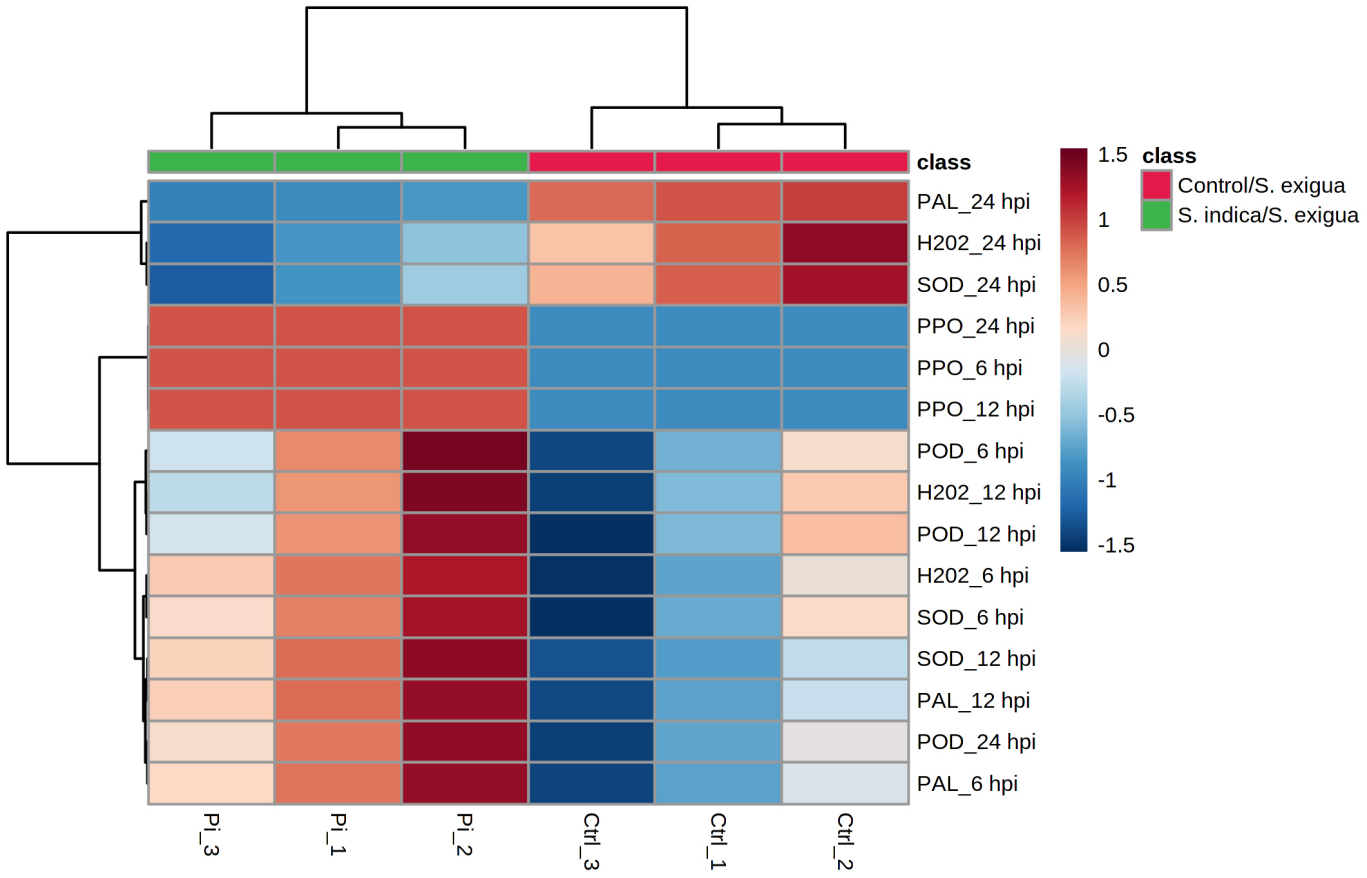
HCA analysis of the onion responses variable to *S. indica* treatment and *S. exigua* infestation represented as a heatmap. Clustering was done by using the Euclidean distance and Ward’s clustering algorithm.

The same data set was used to generate an HCA heatmap (Figure 4). The results revealed that the *S. indica*-treated samples clustered together (Pi_1, Pi_2, and Pi_3), whereas the control samples formed a separate cluster (Ctrl_1, Ctrl_2, and Ctrl_3). The HCA result matched with the PCA results, suggesting the obvious difference in the onion biochemical responses to the *S. indica* treatments under the insect infestation. Out of the fifteen divergent features, 12 clustered together, showing overall higher values in the treated samples and lower in the control. Three variables (PAL_24 hpi, H_2_O_2__24 hpi, and SOD_24 hpi) exhibited overall higher values in the control than the treated plants and formed a separate cluster. Collectively, these data suggested that in the *S. indica*-treated plants, the overall biochemical activities of H_2_O_2_, POD, SOD, PPO, and PAL were upregulated as compared to the untreated control under *S. exigua* infestation. However, at 24 hpi, the activity of PAL, H_2_O_2_, and SOD was found to be higher in the control than in the treated.

### Defense gene expression analysis

**Figure 5:**
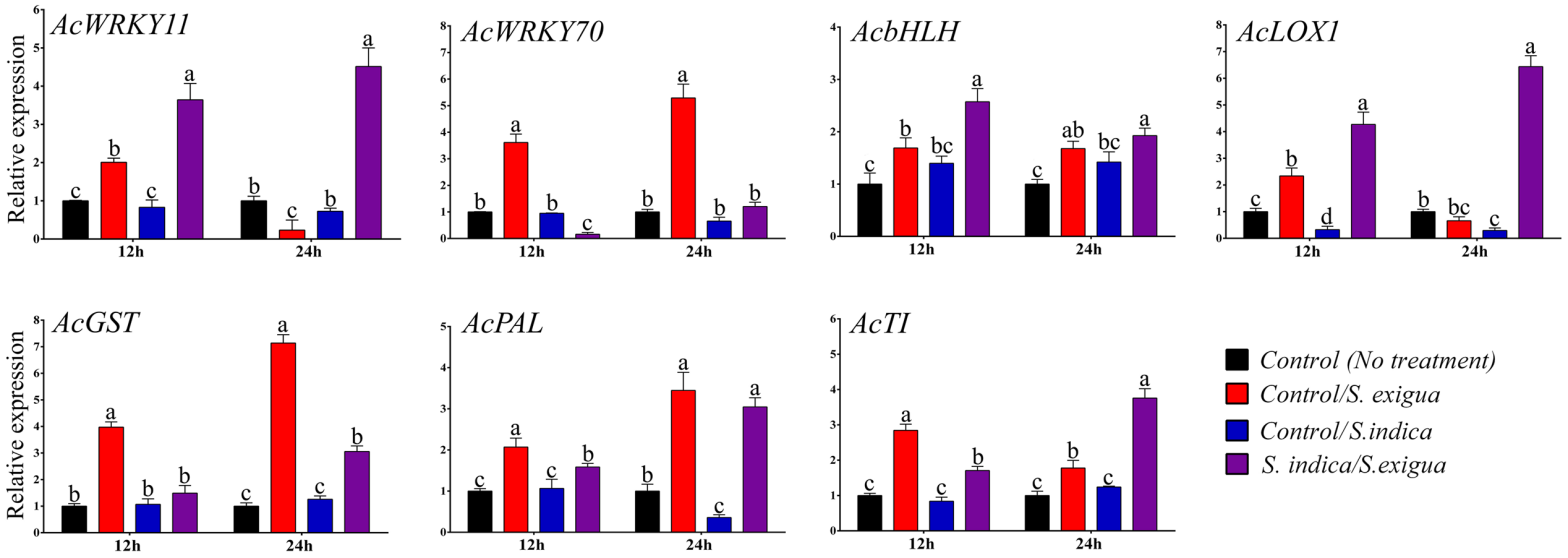
Expression analysis of the selected onion genes in different onion plant groups. Data are represented as mean ± SE and the alphabets indicate the statistical significance computed at *p* < 0.05.

To provide further molecular insight for *S. indica* primed induced resistance against *S. exigua* we have performed the qRT-PCR analysis of seven genes at 12 and 24 hpi (Figure 5). The relevant expression of *AcWRKY11* and *AcWRKY70* were observed to be interconnected to each other. At 12 hpi in control *S. exigua* infested plants, the expression of *AcWRKY11* was insignificant (−1.64 fold) which further drastically down-regulated at 24 hpi (−4.28 fold) as compared to *S. indica* primed *S. exigua* infested plants. While the expression of *AcWRKY70* was opposite of *AcWRKY11* which was highly up-regulated from 12 hpi (3.61fold) to 24 hpi (5.29 fold) in control *S. exigua* infested plants as compared to *S. indica* primed plant, where it got downregulated at 12 hpi (0.15 fold) after *S. exigua* infestation. This trend indicated that the expression of *AcWRKY11* and *AcWRKY70* was modulated more likely by the treatment of *S. indica* in response to *S. exigua* infestation. Furthermore, the expression of *AcbHLH* at 12 hpi (2.57 fold) was significantly up-regulated in the primed plant after *S. exigua* infestation while at 24 hpi there was no significant difference in both the control and primed plant in response to *S. exigua* infestation. Another pair of genes *AcLOX1* and *AcGST* which are the marker genes for JA- and SA- mediated pathways respectively, showed an opposite trend where in primed plants *LOX1*was upregulated at 12 hpi (4.26 fold) and 24 hpi (6.43 fold) while *AcGST*was significant in the unprimed plant at 12 hpi (3.97 fold) and 24 hpi (7.13 fold) in response to *S. exigua* infestation. We have noticed that both *AcWRKY11* and *AcLOX1* were down-regulated in the late stage of infestation in unprimed plants. *AcPAL* showed a significant change in the early stage of infestation (2.07 fold) in control *S. exigua* infested plants, however, there was no significant change in the late stage of infestation compared to *S. indica* primed plants (3.04 fold). A relevant increase in the relative expression level of the onion gene coding for Trypsin inhibitor (*AcTI*) is significantly up at the early stage of infestation in control *S. exigua* infested plants (2.84 fold) while, it was strongly up-regulated in the late stage of infestation in *S. indica* primed plants (3.75 fold). In this temporal expression of the gene in the absence of *S. exigua* infestation, *S. indica* primed plants showed a relative expression in the range of 0.29 to 1.42 folds indicating the defense response might have been triggered by the insect, while the *S. indica* priming attuned the intensity of response in defense pathway-specific manner.

## Discussion

Growth promotion and induction of tolerance to biotic and abiotic stress after *S. indica* symbiosis have been evidenced from several studies in several plant species including onion (Fakhro et al., 2010; Tsai et al., 2020; Roylawar et al., 2021). Such mutualism or symbiosis of plants and microbial communities is an important factor in a natural ecosystem as well as in agriculture. In the present study, we reported that leaf damage by caterpillar *S. exigua* and its weight gain was significantly reduced when fed upon *S. indica*-colonized onion plants. Earlier studies also investigated the colonization of *S. indica* and its impact on insect herbivory. Li et al. (2021) demonstrated that symbiosis of sweet potato and *S. indica* enhanced growth and resistance against *S. litura* which was evidenced by reduced weight gain and less feeding injury. Similarly, *S. indica* colonization of rice roots resulted in increased resistance against rice leaffolder and rice water weevil which showed that *S. indica* enhanced the resistance of plants to leaf and root herbivory by insects (Cosme et al., 2016; Chen et al., 2022). This enhanced resistance is by modulation of antioxidant and defense enzymes/genes in the host as a result of mutual interaction between *S. indica* and the host (Gill et al., 2016).

Biotic and abiotic stresses led to the production of reactive oxygen species in plants. The H_2_O_2_ accumulation was higher at the initial stages and reduced in the late stage in *S. indica* colonized plants in response to herbivory by *S. exigua* than the non-colonized counterpart. It has been shown that the treatment with JA has a positive role in the accumulation of H_2_O_2_ in wounded tomato seedlings (Orozco-Cárdenas et al., 2001). Furthermore, H_2_O_2_ acts as a key signal molecule in oxidative stress signaling and has an adverse effect on insects (Zhu-Salzman et al., 2008; Petrov and Van Breusegem, 2012). This observation suggests that colonization of roots by *S. indica* assisted the host to limit the oxidative stress caused due to insect herbivory. The plant system must scavenge these excess ROS to protect cells from oxidative damage. An earlier study reported a significantly lower level of malondialdehyde (MDA) content in *S. indica*-colonized plants than in non-colonized rice plants after leaffolder infestation (Chen et al., 2022). Similarly, *S. indica* colonization also showed such modulation of ROS after infection by pathogenic fungi in crops like wheat and chickpea (Serfling et al., 2007; Narayan et al., 2017). AM fungi, *R. irregularis* increased resistance against root-knot nematode with a significant reduction in ROS content in tomatoes (Sharma and Sharma, 2017). There is a close relationship between the perception of H_2_O_2_ and the stimulation of defense response to insect herbivory in plants which interferes with feeding, digestion, and nutrient absorption by insects (Maffei et al., 2006; Wu and Baldwin, 2010).

The antioxidant defense is a vital element of the basic metabolism and enables the plant to deal immediately with rapid environmental stress. Anti-oxidative and defense enzymes were induced in onion plants in response to feeding injury by *S. exigua* to protect cells from oxidative damage as well as insect herbivory. Colonization of root by *S. indica* was reported to elevate the levels of these enzymes in several plants in response to biotic or abiotic stress (Sun et al., 2010; Lin et al., 2019; Roylawar et al., 2021). In this study, we demonstrated the elevation of antioxidative and defense enzyme activities in *S. indica-*colonized onion plants. At the early stage of infection higher activities of H_2_O_2_-metabolizing enzymes might have helped in imparting better protection against chewing injury by *S. exigua* in onion plants colonized by *S. indica*. From the obtained results, we assume that *S. indica*-colonized plants were characteristically able to early percept insect herbivory signals leading to triggering of early defense response in the plant. SOD plays a primary role in scavenging ROS by the dismutation of superoxide radicals into O_2_ and H_2_O_2_. Chen et al. (2022) reported the elevation of SOD activity in *S. indica* colonized rice and increased resistance to leaffolder. Similarly, POD enzymes are ubiquitous and are one of the important components in the ROS scavenging system. In contrast, Li et al. (2021) demonstrated that *S. indica* could not increase the SOD in sweet potatoes. It suggests that the response of *S. indica* to anti-oxidative enzymes is host-dependent (Lin et al., 2019; Tsai et al., 2020). Similarly, higher SOD activities were reported in *S. indica-*primed other crops in response to pathogens (Kumar et al., 2009; Roylawar et al., 2021). Its key role in immediate response against insect pests in various plants was reported such as turfgrasses against western chinch bug (Gulsen et al., 2010), tomato, cowpea, and cotton against *S. litura* (Singh et al., 2013), rice against brown planthopper (Wei et al., 2009), groundnut against *S. litura* (War et al., 2012). Further, PAL is an important enzyme in the phenylpropanoid pathway for SA biosynthesis and is known to involve in plant defense against disease and insects. Here, we observed that in the early stage of *S. exigua* feeding PAL activity was higher in *S. indica*-treated plants and then declined in an advanced stage. It indicates that *S. indica* modulated this enzyme response in such a way that plant exhibits immediate higher defense against stress. PAL activity was reported to increase upon insect attack in poplar, maize, *Brachypodium*, cotton, etc. (Hu et al., 2009; Cass et al., 2015; Sha et al., 2015; Lv et al., 2017). Moreover, different biostatistical methods, including PCA and HCA have been used in several studies to screen and analyze the difference between experimental sample sets (test) and controls (Zhang et al., 2020; Sarfraz et al., 2021). In this study, the PCA and HCA results corroborated each other. There was an obvious difference observed in the onion biochemical responses to the *S. indica* treatments under the insect infestation. In the *S. indica*-treated plants, the overall activities of H_2_O_2_, POD, SOD, PPO, and PAL were upregulated as compared to the untreated control under *S. exigua* infestation. In addition, there was an insignificant change in the activities of all the enzymes in control and *S. indica* control treatment while higher activity was in the early stage of feeding in *S. indica/S. exigua* treatment suggesting effective priming of defense response in onion against herbivory by *S. indica*.

In the present study, the expression of genes involved in SA and JA-signalling, like *PAL* and *LOX* was studied. *PAL* is an important gene in the phenylpropanoid pathway for SA biosynthesis, a crucial phytohormone involved in plant defense (Rivas-San Vicente and Plasencia, 2011). *AcPAL* expression was seen to be induced upon *S. exigua* herbivory in onion. Recently, Dixit et al. (2020) reported the higher expression of *PAL* gene is associated with the resistant cotton varieties under *H. armigera* and *S. litura* infestation. Similarly, JA is an important phytohormone involved in plant defense signaling against insects. *LOX* gene, a well-studied JA-pathway gene encoding lipoxygenase enzyme, is involved in the catalyzation of the hydroperoxidation of linolenic acid, an important step in JA synthesis (Bell et al., 1995). Here, *AcLOX1* expression was found to be highly induced in *S. indica/ S. exigua* treatment at both early and late stages of herbivory in onion. Earlier reports also mentioned that root colonization by *S. indica* induces the expression of genes for JA biosynthesis (Lin et al., 2019; Li et al., 2021; Roylawar et al., 2021). It ultimately enhanced resistance to both wound/herbivory and necrotrophic pathogens for diseases. *LOX1* interacts with the other *LOX* genes for the synthesis of oxylipins and regulates the resistance to insect herbivory (Zhou et al., 2014). It was also seen in Maize, that 9-lox are more prominently expressed upon herbivory by larvae of *S. exigua* than 13-lox (Woldemariam et al., 2018).

In the present study, we have studied the expression of two *WRKY* and one basic-helix–loop–helix *(bHLH)* transcription factor (TF). *WRKY* TFs regulate many metabolic processes and are well studied for their role in plants’ defense response against abiotic and biotic stress. Expression of *WRKY11* was found to be induced in *S. indica/S. exigua* treatment in onion. Earlier studies reported that *WRKY11* is involved in bacterial pathogen defense, drought, and salt stress in rice and soybean (Lee et al., 2018; Wang et al., 2018). It was proposed that *WRKY11* induces JA-responsive genes by repressing SA-responsive genes together with *WRKY70* (Journot-Catalino et al., 2006). The higher level of *WRKY11* in our studies may have positively regulated the expression of *LOX1*. This is also evident from the suppression of *WRKY70* in primed plants. We have observed significantly higher expression of *WRKY70* in control/*S. exigua* treatment over others. It suggests that the colonization of *S. indica* fine-tuned the defense response more efficiently. *WRKY70* is an inducer of SA-related genes and a suppressor of JA-related genes (Li et al., 2004). Higher jasmonate level increases plant endurance against different insect herbivore in natural habitat (Machado et al., 2016). *WRKY* TFs are also reported to impart resistance to plants against insects by regulating the mitogen-activated protein kinase (MAPK) pathways (Yao et al., 2020). Our previous study on onion and *S. indica* interaction also hinted at such modulation of *WRKY*s to enhance the resistance of the host plant (Roylawar et al., 2021). Similarly, *bHLH* TFs are involved in JA-mediated signaling in plants (Goossens et al., 2017). The significantly higher-level expression of the *AcbHLH* gene in *S. indica/S. exigua* treatment also assures that the JA-mediated defense pathway is actively getting regulated in *S. indica*-primed plants against insect herbivory. An earlier study in sweet potatoes also reported similar findings where *S. indica* colonization induced the expression of *IbbHLH3* upon wounding by *S. litura* (Li et al., 2021). In tomatoes, the jasmonate-induced *bHLH* gene homolog was found to be associated with terpene biosynthesis ultimately increasing resistance to the *H. armigera* (Cao et al., 2022).

Protease inhibitors affect the availability of nutrients and weaken the development of insect pests. Among them, trypsin inhibitors are well-known and studied protease inhibitors involved in pest resistance (Fürstenberg-Hägg et al., 2013). Here, in onion, we observed induced expression of *AcTI* upon infestation by *S. exigua*. This expression was more in control/*S. exigua* treatment at an earlier stage and later stages expression were higher in *S. indica/ S. exigua* treatment. It was earlier reported that in *S. indica* colonized sweet potato, the activity of trypsin inhibitor was 60% higher than in non-colonized plants upon *S. litura* herbivory (Li et al., 2021). Trypsin proteinase inhibitors’ role in defense response against *S. exigua* was also reported in *Nicotiana attenuate* (Steppuhn and Baldwin, 2007).

## Conclusion

Onion plants colonized with *S. indica* significantly reduced the chewing injury by larvae of *S. exigua* and indeed retarded the growth of larvae. The elevated enzyme activities and defense genes expression at an early stage of chewing damage suggest efficient priming of the defense response in onion by *S. indica*. The primed plants have shown early perception of insect feeding by higher activity of H_2_O_2_. The higher level of activity of PPO and expression of *WRKY11*, *LOX,* and *bHLH* gene in all the time points indicated the prominent role of JA signaling in defense against *S. exigua.* A more targeted approach is needed to dissect the mechanism of this induced resistance where the role of ROS and *WRKY11* will be studied in detail. Our results indicate the potential use of *S. indica* as a biocontrol agent against chewing insects in onion.

## Data availability statement

The original contributions presented in the study are included in the article/Supplementary material, further inquiries can be directed to the corresponding author.

## Author contributions

SG and PR: conceptualization and supervision. PR, KK, and PS: methodology. PR and SN: formal analysis, data curation, and visualization. PR, KK, SJ, and PS: investigation. SG and SJ: resources. PR, SN, and KK: writing – original draft preparation. PR, KK, SJ, SN, and SG: writing – review and editing. SG: project administration and funding acquisition. All authors contributed to the article and approved the submitted version.

## Funding

This work was funded by the Indian Council of Agricultural Research, New Delhi, India (HORTDOGRSIL201800400084). The Article Processing Charge (APC) was funded by ICAR-DOGR, Rajgurunagar (Pune, India).

## Conflict of interest

The authors declare that the research was conducted in the absence of any commercial or financial relationships that could be construed as a potential conflict of interest.

## Publisher’s note

All claims expressed in this article are solely those of the authors and do not necessarily represent those of their affiliated organizations, or those of the publisher, the editors and the reviewers. Any product that may be evaluated in this article, or claim that may be made by its manufacturer, is not guaranteed or endorsed by the publisher.
