# Supplementary material for: Colonization of Serendipita indica promotes resistance against Spodoptera exigua in onion (Allium cepa L.)
Source: Front Microbiol. 2023 Jul 26;14:1190942. doi: 10.3389/fmicb.2023.1190942 (PMC10410256; doi:10.3389/fmicb.2023.1190942)
Supplement: Supplementary file 1 [file Table_1.DOCX]

| **No. of plants tested** | **% Rating (R)** | |
| --- | --- | --- |
|  | **Treated Plants** | **Control Plants** |
| P_1_ | 0.00 | 26.67 |
| P_2_ | 5.56 | 79.17 |
| P_3_ | 0.00 | 60.00 |
| P_4_ | 4.17 | 75.00 |
| P_5_ | 0.00 | 90.00 |
| P_6_ | 41.67 | 66.67 |
| P_7_ | 3.33 | 83.33 |
| P_8_ | 27.78 | 55.56 |
| P_9_ | 6.67 | 94.44 |
| P_10_ | 5.56 | 72.22 |
| P_11_ | 16.67 | 76.67 |
| P_12_ | 37.50 | 22.22 |
| **mean** | **12.41** | **66.83** |
| **Grade** | **1** | **3** |

**Table S1. Evaluation of *Spodoptera exigua* damage on *S. indica* treated onion plants after 24 hrs of infestation**
